# Asymmetric Excitatory Synaptic Dynamics Underlie Interaural Time Difference Processing in the Auditory System

**DOI:** 10.1371/journal.pbio.1000406

**Published:** 2010-06-29

**Authors:** Pablo E. Jercog, Gytis Svirskis, Vibhakar C. Kotak, Dan H. Sanes, John Rinzel

**Affiliations:** 1 Physics Department, New York University, New York, New York, United States of America; 2 Center for Neural Science, New York University, New York, New York, United States of America; 3 Department of Biology, New York University, New York, New York, United States of America; 4 Courant Institute of Mathematical Science, New York University, New York, New York, United States of America; University of Maryland, United States of America

## Abstract

Low-frequency sound localization depends on the neural computation of interaural time differences (ITD) and relies on neurons in the auditory brain stem that integrate synaptic inputs delivered by the ipsi- and contralateral auditory pathways that start at the two ears. The first auditory neurons that respond selectively to ITD are found in the medial superior olivary nucleus (MSO). We identified a new mechanism for ITD coding using a brain slice preparation that preserves the binaural inputs to the MSO. There was an internal latency difference for the two excitatory pathways that would, if left uncompensated, position the ITD response function too far outside the physiological range to be useful for estimating ITD. We demonstrate, and support using a biophysically based computational model, that a bilateral asymmetry in excitatory post-synaptic potential (EPSP) slopes provides a robust compensatory delay mechanism due to differential activation of low threshold potassium conductance on these inputs and permits MSO neurons to encode physiological ITDs. We suggest, more generally, that the dependence of spike probability on rate of depolarization, as in these auditory neurons, provides a mechanism for temporal order discrimination between EPSPs.

## Abstract 2

In order to localize sounds in the environment, the auditory system detects and encodes differences in signals between each ear. The exquisite sensitivity of auditory brain stem neurons to the differences in rise time of the excitation signals from the two ears allows for neuronal encoding of microsecond interaural time differences.

## Author Summary

Animals can locate the source of a sound by detecting microsecond differences in the arrival time of sound at the two ears. Neurons encoding these interaural time differences (ITDs) receive an excitatory synaptic input from each ear. They can perform a microsecond computation with excitatory synapses that have millisecond time scale because they are extremely sensitive to the input's “rise time,” the time taken to reach the peak of the synaptic input. Current theories assume that the biophysical properties of the two inputs are identical. We challenge this assumption by showing that the rise times of excitatory synaptic potentials driven by the ipsilateral ear are faster than those driven by the contralateral ear. Further, we present a computational model demonstrating that this disparity in rise times, together with the neurons' sensitivity to excitation's rise time, can endow ITD-encoding with microsecond resolution in the biologically relevant range. Our analysis also resolves a timing mismatch. The difference between contralateral and ipsilateral latencies is substantially larger than the relevant ITD range. We show how the rise time disparity compensates for this mismatch. Generalizing, we suggest that phasic-firing neurons—those that respond to rapidly, but not to slowly, changing stimuli—are selective to the temporal ordering of brief inputs. In a coincidence-detection computation the neuron will respond more robustly when a faster input leads a slower one, even if the inputs are brief and have similar amplitudes.

## Introduction

In order to localize acoustic objects along the horizontal plane, the nervous system is able to distinguish microsecond differences in the arrival time of sound at the two ears, referred to as interaural time differences (ITDs). Low sound frequencies are the most useful signals for detecting ITDs, and animals with good sensitivity below 1,500 Hz tend to perform best at this perception [1]. In mammals this computation is first performed by medial superior olivary neurons (MSO) in the auditory brain stem. Each MSO neuron receives two sets of excitatory inputs on its bipolar dendrites, with each set activated by one ear. When both excitatory pathways are activated within a narrow time window, the MSO neuron detects the coincident excitatory synaptic inputs and fires action potentials. When the pathways are activated asynchronously, the MSO neurons do not respond. Thus, an ITD response function is the representation of the variation of MSO discharge rate with the relative delay of the two inputs and, therefore, the position of a sound along the horizontal plane [2].

One influential theory holds that ITD encoding is based on an arrangement of axonal delay lines [3]. In this model, the differences in the sound's time of arrival at the two ears is transformed into a spatial map of ITD detecting neurons, sometimes referred to as a “place” code. Thus, an MSO neuron would discharge maximally when a specific ITD is exactly compensated by an internal delay that arises as a consequence of differences in the length of axons that are driven by the two ears. In fact, evidence for this mechanism has been found in birds and mammals [4]–[8]. However, since the discharge rate of many MSO neurons increases over the physiological range of ITDs [9]–[12], this information could also be used to encode the azimuthal position, sometimes referred to as the “slope” code [13]. Additionally, there is evidence to suggest that inhibitory inputs to MSO play a role in tuning the response function within the physiological range of ITDs [11],[14].

In previous models of ITD processing, the propagation time between the ipsi- and contralateral ears to the MSO neurons is implicitly assumed to be equal (excluding Jeffress's internal delay lines). However, MSO neurons are positioned to one side of the brainstem, and the ipsilateral pathway is expected to be shorter than the contralateral. For example, one study has shown in vivo that many superior olivary neurons display longer latencies for the contralateral pathway [15]. Thus, any mechanism that relies on temporal precision must take this into account. We have tested this premise using a novel in vitro preparation that preserves each pathway. Our results support a new mechanistic explanation for the compensation of a longer contralateral response latency, and the positioning of the ITD response function in the physiological relevant range. The mechanism takes advantage of a difference in the dynamics of ipsi- and contralateral excitatory synaptic inputs. Using a computational model, we demonstrate that these asymmetric excitatory synaptic dynamics can significantly alter the ITD responses of MSO neurons.

## Results

**Figure 1:**
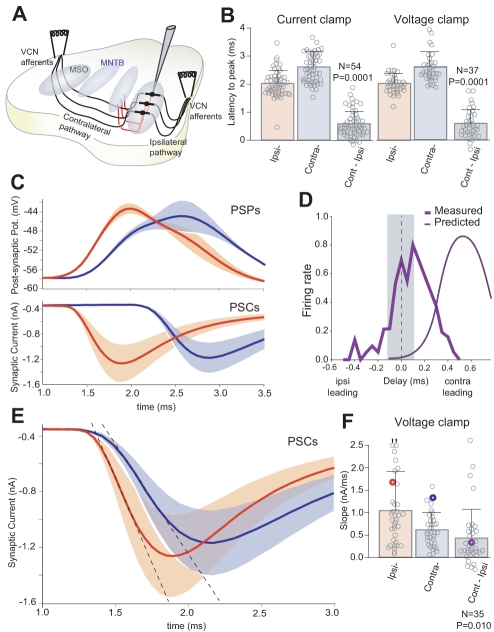
Time difference processing in gerbil MSO in vitro. (A) Schematic of thick slice preparation (500 µm) through the ventral auditory brain stem. Afferent projections from the ipsi- and contralateral VCN are segregated on MSO dendrites. MNTB inhibitory afferents provide contralaterally evoked inhibition to MSO neurons. It is important to recognize that the stimulating electrodes are placed at a position on the auditory pathway that has the same axonal length to the ears, respectively, on each side. (B) PSPs- and PSCs-latency to peak responses (different population sets: voltage clamp data were recorded with intracellular cesium and QX-314). Population's average and standard deviation to show the range of MSO delays. Statistical intervals of confidence were expressed using *t* test with respect to zero for the difference between ipsilateral and contralateral responses on the same neuron (right column: Cont-Ipsi). (C) Average and standard deviation of PSPs and PSCs (average of 50 trials per neuron) for two different sample neurons. The superimposed traces show that the contralateral response occurs at a longer latency. (D) ITD response function in vitro. Bilateral stimulation of VCN afferents elicits action potentials in MSO neurons through coincidence detection of the bilateral PSPs. Gerbil's physiological relevant range (gray bar). Spikes were counted at different stimulation delays to mimic physiological ITD response functions (Measured). Based on the average delay between ipsilateral and contralateral PSPs latency to peak, the ITD response function should be maximal at 580 µs on the contra-leading side (“Predicted” curve is hypothetical, based on bilateral PSP-peak coincidence). The ITD response function peak is close to zero-delay when the bilateral PSPs are summated by the neuron, creating a paradox between the predicted and measured responses. (E) Average and standard deviation of PSCs (average of 50 trials for a sample neuron). The superimposed traces show that the ipsilateral rising slope is steeper. (F) Population data for PSC slopes. Statistical intervals of confidence were expressed using *t* test versus zero for the difference between ipsilateral and contralateral responses on the same neuron (right column: Cont-Ipsi). PSCs in (E) are marked with colored circles. Difference in PSCs is our explanation for the paradox stated in D.

Asymmetries in circuit architecture can have a significant effect on ITD processing. Specifically, the contralateral projections from ventral cochlear nucleus (VCN) to MSO are longer than those from the ipsilateral side (Figure 1A, difference in afferent lengths between ipsilateral VCN to MSO and contralateral VCN to MSO ≈2.45 mm; Paul Nakamura and Karina Cramer, personal communication). To measure this difference functionally we used a thick brain slice preparation from gerbils that preserves the afferent pathways to the superior olivary complex (Figure 1A; see Methods). Whole cell recordings were obtained from MSO neurons while activating each pathway at the same anatomical position on each side; the pathway between the stimulation point and the cochlea, which is eliminated in this preparation, is assumed to be identical for each side (Figure 1A). We first found that the response latency did, in fact, differ between the two pathways. An analysis of evoked postsynaptic potentials (PSPs) and currents (PSCs) showed that the latencies to peak of contralateral responses were on average about 500 µs longer than those of ipsilateral responses on the same recorded neuron (Figure 1B,C; average differences in latency to peak for PSPs: 573±62 µs, *n* = 54; for PSCs: 589±81 µs, *n* = 37, see Methods section). This difference was apparent on a cell-by-cell basis because the difference of latencies (contralateral - ipsilateral) was significantly different than zero (see gray bars in Figure 1B).

In presenting the following experiments, we refer to the in vitro inter-stimulus time difference as ITD. Thus, if threshold were to depend solely on PSP amplitude, then the measured disparity in PSP latencies would predict that the peak ITD response would occur when the contralateral PSP leads by approximately 500 µs (Figure 1D; predicted, thin curve). This ITD value is sufficiently large that the response function would fall largely outside of the physiological range for gerbils, which is ±130 µs [16]. In contrast, we found ITD response functions in which MSO firing rate was maximal when bilateral stimuli were delivered with smaller delays of ≈100 µs (Figure 1D; measured, thick curve). This finding suggests that an intrinsic integration mechanism must compensate for the longer contralateral path.

MSO neurons are exquisitely sensitive to the rate of depolarization. Therefore, in order to understand the integration of subthreshold bilateral inputs that lead to a spike, we examined the dynamics of synaptic inputs. Our starting assumption had been that synaptic properties are identical for each of the two excitatory inputs to MSO. We examined this assumption by measuring the rising PSP slopes because their time scale is within the same range as the coincidence detection window as manifested by the width of the ITD response function (i.e., 0–250 µs). Ipsilaterally evoked PSCs had significantly steeper rising slopes than contralateral PSCs (Figure 1E,F) (ipsilateral: 1.04±0.15 nA/ms, contralateral: 0.62±0.06 nA/ms; *p* = 0.01, *n* = 35). This difference was apparent on a cell-by-cell basis because the difference of PSC slopes (contralateral - ipsilateral) was significantly different than zero (see gray bar in Figure 1F). This result was independent of stimulus amplitude in all tested neurons (see Figure S1). The differences in the slopes of the PSCs could compensate, in part, for the disparity in delay between the two pathways. Our computational model (below) showed that even a modest asymmetry in rising slopes could shift the ITD response function from its hypothetical position (based on latencies to peak) to the observed location in the in vitro experiment (Figure 1D).

**Figure 2:**
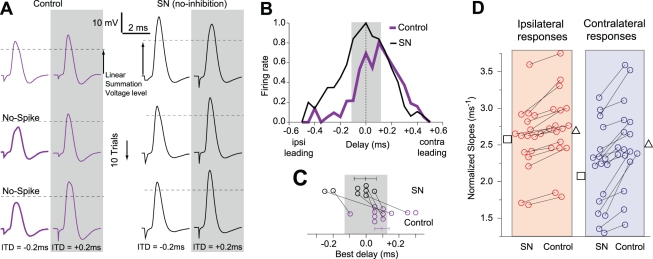
Effect of asymmetric PSPs and EPSPs in setting best ITD position. (A) Voltage time courses of somatic depolarization under bilateral stimulation at different delays (example cell, P21, T = 32°C). Each column shows three representative trials for a specific ITD value. The dashed lines show the spike identification-threshold for our experiments, and these were used to plot the ITD functions shown in (B). When glycinergic inhibition was blocked (SN), firing rate increased for ipsilateral leading ITD stimulations (compare column 1 (1 spike in 3 trials) with column 3 (3 spikes in 3 trials)). (B) ITD response function for control case and when blocking synaptic inhibition (SN) for 10 stimulation trials. Shaded area is the physiologically relevant range (±130 µs). (C) Best ITD response for different cells under control and with SN. Predicted best ITDs for control should be around 580 µs on average. Instead our data show that best ITD responses are closer to a delay of zero. When inhibition is blocked the best ITDs shift towards ipsi-leading responses (*N* = 12, postnatal days 17–25; disconnected points are from experiments that were performed either in control or under SN only). (D) Increase in slope when inhibition is present. Slopes for normalized EPSP (SN, no-inhibition) are: ipsi 2.61±0.11 ms^−1^(square), contra 2.21±0.14 ms^−1^ (triangle); *p* = 0.031, *n* = 17. When inhibition is present (Control) PSP slopes are: ipsi 2.71±0.12 ms^−1^, contra 2.49±0.10 ms^−1^; *p* = 0.064, *n* = 17. Stronger effect of the inhibition on contralateral responses (contra-inputs difference 0.28 ms^−1^, *p* = 0.05, *n* = 17; ipsi-inputs difference 0.10 ms^−1^, *p* = 0.05, *n* = 17). Bilateral EPSPs are even more asymmetric than the PSPs, suggesting a possible explanation for why best ITDs are more shifted to the ipsi-leading side under SN (see below model results, Figure 5).

To determine how this asymmetry in excitatory synapse kinetics might compensate for the differences in path length, it was first necessary to determine the contribution of synaptic inhibition. To address this issue, we obtained ITD response functions under current clamp (CC), before and after application of a glycine receptor antagonist, strychnine (SN). As shown in Figure 2A and 2B, when synaptic inhibition was present (control), the maximal firing occurred for contralateral leading stimulation, consistent with in vivo recordings [9]–[12]. When synaptic inhibition was blocked (Figure 2A and 2B, SN) the maximal firing rate was close to zero ITD, also consistent with an in vivo study [11]. We calculated the ITD at which peak firing probability occurred (“best ITD”) for the population of recorded neurons (Figure 2C) and found that under control conditions the peak was at 105±35 µs (contra-leading), while under SN conditions it was at −62±38 µs (ipsi-leading). Therefore, the effect of synaptic inhibition was to shift ITD tuning towards contralateral leading stimuli. Since this shift is in the wrong direction to compensate for the longer contralateral path, we next considered the role of asymmetric excitatory responses.

In the presence of inhibition (control), the ipsilaterally evoked normalized PSP slope was 2.71±0.12 ms^−1^ and the contralateral slope was 2.49±0.10 ms^−1^ (Figure 2D). When inhibition was blocked (SN), evoked EPSP slopes were significantly different between ipsi- and contralateral responses (ipsilateral: 2.61±0.11 ms^−1^; contralateral: 2.21±0.14 ms^−1^, see Figure 2D and also Figure S2). Blockade of glycinergic inhibition increases the differences in the PSP slopes. More specifically, inhibition always increases the slope (Figure 2D, from squares to triangles), but more so for the contralateral responses (Figure 2D, right column). Such steepening occurs for either fast or slow inhibitory synaptic conductance transients (see Figure S3 for theoretical support). In the fast case (Figure S3, left), the decaying brief IPSC coincides with rising EPSC and the summed current therefore rises faster than the EPSC alone. The effect is stronger on contralateral inputs because the IPSC will more fully decay during the EPSC rise. In the slow case, the IPSC transiently reduces the effective time constant, accelerating the rise although less dramatically than does a fast IPSC (Figure S3, right). The effect is stronger for contralateral inputs partly because integration of slower inputs is affected more by time constant changes (leakage matters in addition to capacitive integration). Another major contributing factor related to active currents is explained below with our model. Thus, we confirmed that synaptic inhibition reduced the effect of shifting the ITD response function towards zero ITD, and leads us to suggest that the compensation arises from the excitatory asymmetry described above (Figure 1).

**Figure 3:**
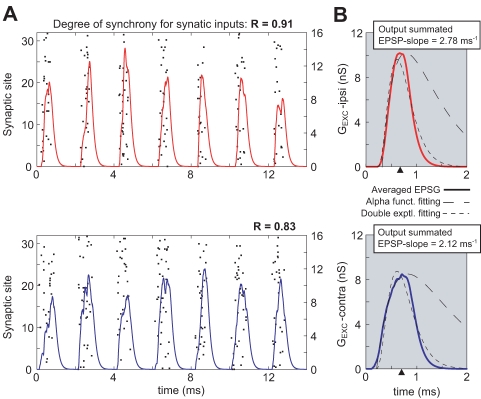
Example of composite EPSGs for an input train (500 Hz) using an idealized auditory nerve fiber model (sinusoidally modulated Poisson rate for mini-EPSG times). It is less realistic than Carney's model (1993)[18] that we used for Figures 4 and 5. (A) Raster plots with event times for ipsilateral excitatory inputs (red) and contralateral excitatory inputs (blue). Superposition of composite EPSGs for the two cases. Mini-EPSGs are alpha functions with time constant of 0.1 ms (see Methods). The difference in vector strength (degree of synchrony) between the events dictates the shape of the composite EPSGs. (B) Average over 25 cycles of composite EPSGs for the two different input cases. In the two cases a function (dashed) was fitted to the average PSG. Alpha function is the simplest description of PSGs (long dashes); it fits the rising phase but not the falling phase. For our summated EPSGs a more complicated function (see below) matches better the envelope or composite EPSG (in the case of excitation). EPSGs integrated through our MSO neuron model give EPSPs rising slopes that are steeper when vector strength is larger (normalized ipsi-EPSP's slope 2.78 ms^−1^, contra-EPSP's slope 2.12 ms^−1^). Composite EPSGs in (B) are fitted by functional form that is proportional to (1−(exp(−(t−t_0_)/τ_rise_))).3.(exp(− (t−t_0_)/τ_decay_)). Ipsilateral inputs (red): τ_rise_ = 2.5, τ_decay_ = 0.14. Contralateral inputs (blue): τ_rise_ = 2.8, τ_decay_ = 0.18. For the alpha functions fits, for ipsilateral inputs (red): τ = 0.48; for contralateral inputs (blue): τ = 0.69. All τ values in ms. Note: the position of the peak of the summated EPSP does not depend on vector strength (see dark triangles in B).

How can such a small asymmetry in EPSP slope influence ITD sensitivity in MSO neurons? We addressed this question by using a computational MSO neuron model that was driven by bilateral trains of excitatory and inhibitory inputs temporally modulated with a periodic function representing VCN responses to pure tone stimuli. Each cycle's composite input was generated from many small excitatory postsynaptic conductances (EPSGs) with statistics that depended on VCN afferent activity that varied with sound frequency and amplitude (see Methods; [17],[18]). Figure 3 shows a simplified version of the simulated MSO inputs to illustrate the variability of the composite EPSGs and integrated EPSPs due only to the jitter on the mini-EPSGs time release. Here, we exclude firing rate modulation throughout the sinusoidal input's cycles, although it is employed in the detailed model used for the simulated ITD functions. Using only differences in vector strength of the simulated inputs from the VCN arriving to each dendrite of the MSO neuron model we modeled differences in rising slope of the bilateral EPSPs (Notice: without delaying the composite EPSP peak, see triangles in Figure 3 for EPSG peaks). These differences led to shifts in the ITD response function that are large enough to compensate for the longer contralateral input pathway. For a given EPSG input, the evoked EPSPs and spike threshold will be determined by the active currents. In MSO and other auditory processing centers, a *low threshold potassium current* (I_KLT_) exerts control on spike threshold [19]–[21]. This fast I_KLT_ imposes a filtering effect on the synaptic inputs allowing only steep EPSG slopes to evoke an action potential [22],[23]. Therefore, steeper EPSGs are more likely to trigger spikes, even when shallower EPSGs may have greater amplitude, as is shown in our simulations.

**Figure 4:**
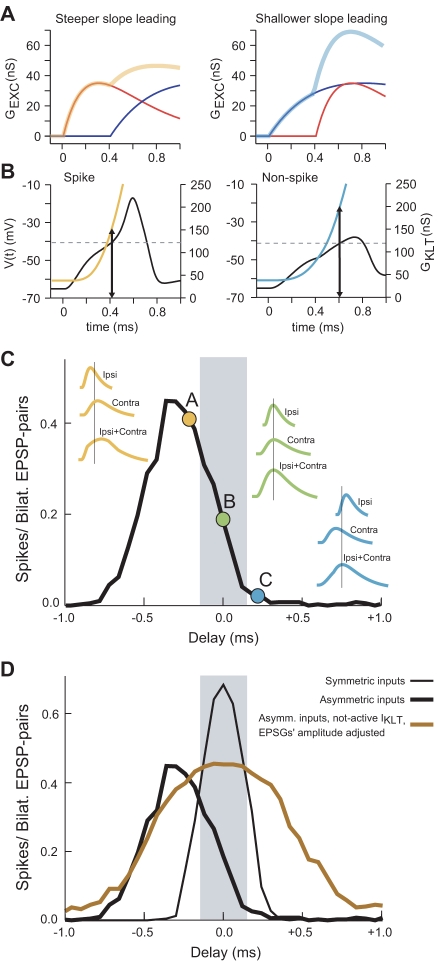
The differential dependence of spike probability on EPSP's slope disappears when IKLT is disabled. (A) Pairings of EPSGs with different time constants and different temporal order. Left: steep EPSG (red curve) arrives earlier than shallow EPSG (blue curve). The summated EPSG pair (steeper then shallower; ipsi then contra) leads to a spike. Right: same EPSGs with reverse order but same time difference does not generate a spike. Inputs' delays different than zero in which steeper EPSP is arriving earlier will generate more spikes than the inputs' delays with the opposite order of arrival time. (B) Voltage time courses (black curves) for the case of spike and non-spike illustrated in (A). G_KLT_ (orange and light blue curves) corresponding to the same two temporal ordering cases. Amount of G_KLT_ recruited (arrows) for the same voltage amplitude in the two time order cases. The deflection in voltage prior to spike generation is indicated by a dashed line. Notice: the amount of G_KLT_ is higher in the case of the shallower EPSP leading; this yields the same proportional increase for I_KLT_ since driving force is the same in both cases. For a given pair of EPSPs, the steeper EPSP arriving first recruits less G_KLT_ and therefore decreases spike threshold level. (C) ITD function shifting due to asymmetric EPSPs. Simulation for ITD detection with asymmetric EPSPs using Carney's (1993)[18] synaptic input model (bilateral EPSP trains at 500 Hz). Colored time courses: schematics of the combination of EPSP-pairs (without spike; vertical lines are to identify relative time delays), three cases: ipsi-leading (steeper then shallower, A), perfect coincidence ITD = 0 (B), and contra-leading (C). Pairs in (A), (B), and (C) cases have different rising slope. Maximum firing rate is generated for the steepest rising EPSP-pair (steep ipsi-EPSP leading) as explained in (A). (D) Differential dependence on EPSP's slope disappears when I_KLT_ is disabled. Thin black curve: ITD function for bilaterally symmetric inputs (vector strength, *R* = 0.9). Thick black curve: ITD function for asymmetric inputs (R_IPSI_ = 0.95, R_CONTRA_ = 0.63), same curve as in (C). Thick brown curve: G_KLT_ is frozen at its rest value preserving the model's passive properties (I_KLT_ behaves as a passive current). For comparison, EPSGs are adjusted to give the same spike probability as in the previous case.

When bilateral subthreshold inputs arrive at an MSO neuron, there is a higher probability of eliciting a spike when the steeper EPSG arrives first. Figure 4A shows how a pair of EPSGs, one fast and one slow, can produce a very different outcome, depending on their order of arrival. When a faster input arrives first this will enable spike generation (Figure 4A and 4B, left side). When a slower input arrives earlier it leads to a slower rising EPSP that recruits more I_KLT_ conductance, which hinders spike generation even though a faster EPSG arrives subsequently (Figure 4A and 4B, right side).

To show the essence of the ITD response function shift due to the asymmetry in the kinetics of the excitatory inputs we delivered inputs to the model with different vector strength (Figure 3) and calculated their probability to evoke spikes for different input delays (ITD response function, Figure 4C,D). If the contralateral composite EPSP was slower-rising, the bilateral combined EPSP had different rising dynamics when the ipsilateral inputs led than when the contralateral inputs led (Figure 4C, EPSPs schematics). Consistent with previous findings [21],[24]–[26], the shallower-leading combined EPSP was associated with a lower probability of firing. Therefore, the ITD function shifted towards the ipsilateral leading side (Figure 4C). The asymmetry in firing rate probability caused by an asymmetry in inputs' rising slopes is due to the voltage-dependence of I_KLT_ conductance. We explain this (Figure 4D) by showing that with the same set of bilateral asymmetric EPSPs that generate a shift of ∼400 µs (Figure 4D, thick black curve), the shift of the ITD's response function disappears (Figure 4D, brown curve) if we fix the I_KLT_ conductance at its resting value, in order to maintain the neuron model's time constant and input resistance intact.

**Figure 5:**
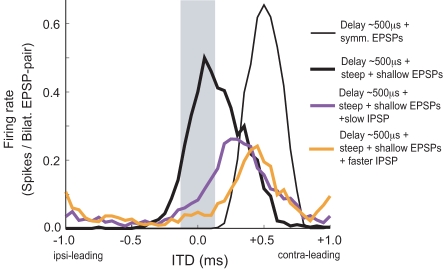
Model prediction of ITD response function using experimental data. *R* is the vector strength of the monolateral presynaptic input. Bilateral input trains of 500 Hz. Thin black curve: simulated ITD function if EPSGs are symmetric. Longer contralateral delay of 500 µs, as our experimental data show. If the pre-synaptic afferents are symmetric, each with *R* = 0.90, the rising phase (only) of the summated EPSGs was fit by an alpha-function with τ_exc_ = 0.14 ms (EPSP-slope = 2.75 ms^−1^). Thick black curve: same contralateral delay as the previous case but with asymmetric excitatory inputs. Contra- is shallower than ipsi-, slope-ipsi-EPSP = 3.1 ms^−1^ (*R* = 0.93, mini-EPSG-τ_exc_ = 0.1 ms), slope-contra-EPSP = 1.6 ms^−1^ (*R* = 0.60, EPSG-τ_exc_ = 0.1 ms); for EPSP shapes see also Figure S4. Violet curve: adding contralateral inhibition preceding excitation by 0.2 ms shifts ITD function towards contralateral leading inputs; slow IPSPs (IPSP-slope = 0.6 ms^−1^ (*R* = 0.5, IPSG-τ_inh_ = 0.4 ms)). Orange curve: faster IPSP than previous case creates larger shift (IPSP-slope = 0.95 ms^−1^ (R = 0.7, IPSG-τ_inh_ = 0.4 ms)).

We next asked whether the asymmetry in the excitatory inputs could compensate for an intrinsic input delay of ≈500 µs as measured in our in vitro preparation. The simulations showed that the integration of hypothetical symmetric EPSPs led to an ITD response function that was shifted to the contralateral leading side due to the intrinsic contralateral axonal delay (Figure 5, thin black curve). When asymmetric EPSGs were introduced in the model to generate EPSP slopes similar to those found in our experiments, the ITD function shifted towards the ipsilateral-leading direction due to the favorable response when a steep EPSP occurs first (Figure 5, thick black curve).

Our experimental data were consistent with this theoretical explanation: most of the neurons displayed this asymmetry in excitatory inputs. Thus, when we subtracted contralateral slope from ipsilateral slope for each individual neuron, the average difference was 0.69±0.18 nA/ms for EPSCs and 0.40±0.12 ms^−1^ for normalized EPSPs. Inclusion of synaptic inhibition made the simulated EPSPs less asymmetric. The hyperpolarization from inhibition transiently reduced I_KLT_. The reduction of this conductance would no longer favor spike generation when fast EPSPs are followed by slow EPSPs. The ITD response function was reduced on the ipsilateral-leading side, giving the appearance of a shift towards the contralateral-leading side (Figure 5, orange and violet curves), as observed experimentally in vitro (Figure 2B) and as reported previously in vivo [11],[14].

## Discussion

Our experimental and computational findings identified key biophysical factors that, together, position the ITD response function in the biologically relevant range. We first confirmed the presence of an internal delay of the longer contralateral pathway (Figure 1B). In itself, this would cause MSO neurons to fire mostly to ITDs with stimuli having large contralateral leading stimuli that are outside the physiological range. Our experimental and computational results suggest a novel excitatory synaptic mechanism that could compensate for the disparity in path length. An asymmetry in the slopes of EPSPs (Figure 2D) can bias the ITD coding in favor of the ipsilateral-leading inputs (Figures 4 and 5), and this repositions the ITD function within the physiological range, as found in vivo [9]–[12].

The presence of a fixed internal latency difference will affect all models of ITD processing. Jeffress [3] assumed tacitly that the two paths were equal in length except for the small differences along one spatial axis of the encoding nucleus. Others have suggested that the shorter path length from the ipsilateral ear is compensated by an additional span of axon (e.g., see schematic in [2]), or a difference in myelination between the two pathways [27]. If the difference in path length to MSO for the gerbil is ≈2.45 mm (Paul Nakamura and Karina Cramer, personal communication), then our electrophysiological measurements of response latency difference of 500 µs would yield a propagation speed of 4.9 m/s. Thus, it appears that there is an internal latency difference to gerbil MSO that is not compensated for by an axonal property. It is this functional characteristic that must be addressed if MSO neurons are to encode ITDs in the physiological range (±130 µs; [16]).

Our electrophysiological measurements indicate that the rising PSP slope is larger for the ipsilateral input to MSO neurons on a cell-by-cell basis (Figures 1 and 2). The functional implications for this finding are illustrated in a computational model which demonstrates that this property can compensate for the aforementioned difference in path length (Figure 5). The general principle, which is that the rising slope of an EPSP determines the probability of firing, is consistent with findings from other systems [21],[25],[26],[28]. Here, we have adapted this principle to resolve the general problem of compensating for different input latencies due to path length.

How might the EPSP asymmetry arise? In the model we allowed for more jitter in the arrival times of identically shaped unitary (minimal) EPSPs on the contralateral side, which slowed the rise of the composite EPSPs. This idealization, for demonstrating plausibility in the context of our point neuron model, could be elaborated and explored in a neuron model that has bilateral dendrites with cable properties [29]. Many alternative mechanisms are also possible. Bilateral differences in dendritic morphology or the dendritic positioning of excitatory terminals could also lead to an asymmetry in the rising slope of composite EPSPs [30],[31]. Although longer electrical distances would promote broadening of composite EPSPs in a passive dendrite, I_KLT_ in the dendrites can reduce the effect by shortening the tail of EPSPs as they propagate toward the soma in MSO neurons and cable models [32]. Alternatively, the distribution of active currents could modulate the dendritic integration of synaptic inputs. For example, dendritic sodium channels are able to selectively boost EPSPs on one dendrite, and this would modify their rising slope (cortex: [33]).

It is important to consider the in vivo time scale of inhibition and excitation because it will determine the temporal integration window and the extent to which ITD curves will be affected by the mechanisms described above. It is possible that the time scales in vivo are faster than in the brain slice because a cell is in a high conductance state (e.g., many more active inputs as compared to brain slice). In addition, the degree of afferent synchrony could have been unnaturally high in our preparation because the stimulus simultaneously recruits all VCN afferents to MSO. However, the model demonstrated that the effect of slope is robust when implemented with vector strength values that have been reported in vivo (Figure 3; using model from [18]). Since we also showed that synaptic inhibition somewhat counteracts the shifting effect of the asymmetric excitation, it is important to consider its kinetics. The time scale for inhibition has only been studied in vitro, and even the fastest IPSPs have either been recorded from animals between 12 to 25 postnatal days [34], or at room temperature [35]. Interestingly, we found that while the magnitude of the inhibitory effect depends on IPSP time scale, it is likely to play an important role in ITD coding no matter what the actual time scale value turns out to be (Figure 5; Figure S3).

The faster rising EPSPs that were elicited by ipsilateral afferents could overcome the penalizing effect of a rapidly activating outward current like I_KLT_ (Figure 4B). Many previous reports have demonstrated a robust effect of I_KLT_ on the integration time of EPSPs [19],[20],[28]. In this study, we applied this property to anatomically independent bilateral inputs and demonstrated computationally that I_KLT_ influenced the ITD function.

Together, our findings lead us to propose a general principle. Passive neuronal integration to a threshold would not distinguish the temporal ordering in inputs that may have different rising slopes. Subthreshold dynamic negative feedback such as I_KLT_ (comparably as fast as integration) will bias the integration. Firing will be favored when the steeper-rising input occurs first. Inhibition, by deactivating the negative feedback, can reduce the bias. The competition between these two effects in the MSO, leads to a positioning of the ITD response function with its slope in the physiological range, as seen in vivo [11]. Thus, the synaptic property compensates for the intrinsic latency disparity. Time-difference encoding could exploit these mechanisms in this extremely short window of integration time (130 µs) or, more generally, in other windows where the biophysical components and time scales are appropriately matched. Generalizing, we propose a novel neuronal mechanism for temporal order selectivity. Subthreshold dynamic negative feedback can increase a neuron's firing probability to segregated subthreshold inputs when faster ones precede slower ones, even if the slower one is of similar or larger amplitude.

## Methods

### Experiments

All protocols were reviewed and approved by New York University Institutional Animal Care and Use Committee. Postnatal day (P) 17–25 gerbils (Charles River) were used to generate thick (450–500 µm) horizontal slices (*N* = 91) from the ventral auditory brainstem. Each slice contained the MSO nucleus, the medial nucleus of the trapezoid body (MNTB), and the lateral nucleus of the trapezoid body (LNTB). Animals were deeply anesthetized (chloral hydrate, 400 mg/kg), perfused intracardially with artificial cerebrospinal fluid (ACSF: 123 mM NaCl, 4 mM KCl, 1.2 mM KH_2_PO_4_, 1.3 mM MgSO_4_, 24 mM NaHCO_3_, 15 mM glucose, 2.4 mM CaCl_2_, 0.2 mM ascorbic acid; pH = 7.35 after bubbling with 95% 0_2_/5% CO_2_) at 32°C. The brain was then dissected free in 32°C oxygenated ACSF, and one horizontal slice was obtained with a Leica vibratome. The slice was incubated at 36°C for 20 min, and at 22°C for 1 h before being transferred to the recording chamber where oxygenated ACSF was perfused at a rate of 2 ml/min at 32°C; temperature was regulated by L&N temperature controller.

The afferents arising from both VCNs were visualized as compact bundles. Thus, ipsilateral and contralateral bundles were stimulated at the site of their origins with bipolar tungsten electrode and stimulation was delivered by two stimulus isolation units (Dagan). The distance between the MSO and the two stimulation sites was approximately 0.5 mm for the ipsilateral pathway and 1.5 mm for contralateral pathway. Whole cell current-clamp recordings were obtained mostly from medial and dorsal MSO neurons (Axoclamp2A). The recordings and stimulation were computer driven (Windows XP) through Labview software (National Instruments). The neurons were visually identified using infra-red differential interference contrast (IR-DIC) microscopy (Olympus). The internal patch solution contained (in mM) 127.5 potassium gluconate, 0.6 EGTA, 10 HEPES, 2 MgCl2, 5 KCl, 2 ATP, 10 phosphocreatinine (Tris salt), and 0.3 GTP (pH 7.2) in the case of CC protocol and (in mM) 127.5 cesium gluconate, 0.6 EGTA, 10 HEPES, 2 MgCl2, 5 KCl, 2 ATP, 5 QX-314,10 phosphocreatinine (Tris salt), and 0.3 GTP (pH 7.2) in the case of voltage clamp (VC) protocol. In order to block synaptic inhibitory inputs, we used SN in CC experiments and SN and bicuculine to block glycinergic/gabaergic inputs in VC experiments.

EPSPs in CC and EPSCs in VC were recorded when single square pulses repeatedly (20 Hz) of 25–50 µs were delivered via the stimulating electrodes to initially evoke minimum amplitude responses, maximum amplitude subthreshold responses, and subthreshold-unilateral/suprathreshold-bilateral responses. High stimulus currents (0.5 to 10.0 mA) and short pulse durations (25–50 µs) were used to avoid the overlap of stimulus artifact with evoked responses. The data were analyzed following these basic criteria: slopes of the rising phase (20% to 80%) of the responses, for unilateral stimulations. For all the parameters that were measured for bilateral stimulations responses (i.e., peak-delay, slope), the intervals of confidence (*p* values) were computed using *t* test over the difference between ipsi- and contralateral responses on the same neuron. All data variability is expressed in standard deviation. In addition, 100 to 500 Hz stimulus trains of 10 stimuli were applied (total number of spikes per train delay were counted) to generate ITD tuning response function. A minimum of four trials were run to get a smooth ITD response function. In the case of CC data the slopes of PSPs and EPSPs were computed when bilateral responses were similar in amplitude, to avoid differential effect of active currents, and were normalized to decrease population variability due to biophysical heterogeneity among neurons.

### Simulations

We used a computational model of MSO neurons based on the parameters described by Rothman and Manis (2003)[36] for a point VCN neuron [36]. We chose a membrane time constant of 0.3 ms, similar to the one reported for MSO neurons after P20 [20]. Bilateral input trains with different delays were created by injecting currents (conductance based synaptic-like currents) such that the trains of EPSPs consisted of composite minimal EPSPs (32 or 64 minimal EPSPs were used to create a ∼8 mV composite EPSP; more EPSGs were used for higher input frequencies (1.1 KHz) to generate a smooth voltage time course). Minimal EPSGs had fixed form: alpha functions with time constant τ_syn_ of 0.1 ms for excitation and 0.4 for inhibition, scaled to have specified area and peak proportional to 1/τ_syn_. Different minimal EPSG statistics led to different slopes and half-widths, which are summed in order to create the composite suprathreshold EPSGs (see Figure S4). These EPSPs have envelopes resembling alpha functions with time constants that ranged from 0.1 to 0.8 ms [17]. This range of (in vivo based) EPSP time constants was slightly faster than those obtained from our experiments because our recordings were made at 32°C and the simulations were performed at 37°C. The same results were obtained using values of rising EPSP slopes from our experiments at 22°C as well as the kinetics of our computational model, to eliminate any temperature effect.

The asymmetry in simulated EPSP kinetics was modeled by varying the jitter of unitary events. The amount of jitter was based on the observed variability in EPSC amplitudes, slopes, and half-widths obtained in our brain slice recordings. ITD functions were created from bilateral EPSP or PSP trains (40 cycles) at frequencies ranging from 250 to 1,100 Hz. A minimum of 10 trials (per ITD) were run to get a smooth ITD response function.

The differential equations of the model were integrated numerically using fourth-order-Runge-Kutta scheme with a time step between 1 and 0.25 µs; refining the time step did not lead to noticeable differences in the computed solutions.

In all the simulations the contralateral inhibitory input leads the contralateral excitation by 0.2 ms. This time difference was imposed between the peak of the composite IPSPs and the composite EPSPs from the contralateral input side. The result in Figure 4 showing that inhibition shifts the ITD response function towards contralateral leading side holds even for bigger delays between contralateral inhibition and excitation (unpublished data).

## Supporting Information

Figure S1 **PSP-rising-slope and -peak-delay versus PSP response amplitude for different VCN-afferents stimulation strength.** (A) Examples of individual neurons showing that asymmetry of rising slopes between bilateral inputs remains, when the response amplitude changed (top: current clamp experiment, P21; bottom: voltage clamp experiment, P23). (B) The distribution of peak latencies for PSP responses is almost flat for the range of subthreshold PSPs and PSCs (top and bottom plots, respectively, same neurons in A). (C) Summarized data for 14 experiments in current clamp and 16 in voltage clamp configuration (see Methods). Left: rate of change for slope at different amplitude responses is similar between sides, supporting the result that when PSP slopes are asymmetric between sides they will remain asymmetric through the subthreshold range. Right: rate of change of latencies is close to zero for different response amplitudes. Even though the individual monolateral response has jitter, the ITD response function is the average latency of the PSP responses and this is statistically unchanged with the amplitude of the response.
(0.63 MB EPS)

Figure S2 **Subthreshold evoked-PSPs stimulating the afferents of the VCN onto the MSO neurons on our thick slice preparation.** (A) In most of the neurons recorded ipsilateral responses were bigger in amplitude than the controlateral; for this reason all the data in the paper are normalized by amplitude to avoid bias on the comparison between contralateral and ipsilateral rising-phase slopes. Cells had been recorded from both olives on the same slice, keeping the stimulating electrodes on the same location to avoid possible sources of asymmetries due to the stimulation artifacts. Time courses show that PSPs are a composition of many synaptic mini-PSPs released with a particular distribution of locations and/or timing. Dendrite morphology could play a role on the asymmetry in EPSPs' shapes. Superposition of ipsilateral and contralateral responses shows a consistent trial-to-trial difference on rising phase. (B) When inhibition is blocked voltage time courses show that EPSPs are a composition of many synaptic mini-EPSPs released with a particular time distribution. Decay phase is similar between the bilateral responses dictated by “effective” membrane time constant (combination of active currents) and integration properties of bipolar dendrites. (C) Top row: slopes versus halfwidth for PSPs evoked from contralateral and ipsilateral stimulations. Ipsilateral responses are steeper than contralateral, with similar halfwidth for both responses. Passive propagation of EPSP through asymmetric dendrites is not enough to explain the difference on asymmetric responses recorded at the soma compartment due to the similarity in halfwidths. Normalizing the halfwidths and slopes by the corresponding individual PSP amplitudes show consistent results supporting the fundamental observation that the bilateral asymmetry is intrinsic in the neurons independently from the trials and response amplitude. Bottom row: same as the top row but now glycinergic inhibition is blocked. Note: EPSP shapes are similar to the ones obtained from the model when we simulate the synaptic excitatory conductance as population of inputs with different jitter for each bilateral input (see also Figure 3 and Figure S4). In vivo EPSP slopes could be smaller than the ones recorded in our experiments due to the fact that in our preparation there is a high degree of synchronicity due to the simultaneous stimulation of the VCN bundle. Larger ipsilateral response will strongly support our results. For simplicity and taking a conservative position we will use for the modeling equal amount of conductance between bilateral inputs. Only for some schematics will we use the same amplitude for bilateral inputs.
(0.85 MB EPS)

Figure S3 **Increase in slope due to fast and slow (extreme values) inhibitory conductance (from our parameter study this effect is seen if 0.2 (ms)<δ for τ_inh_ = 0.1 (ms) and 0.75 (ms) <δ for τ_inh_ = 1.0 (ms)).** EPSPs with a shallower slope are more affected by synaptic inhibition for a large range of inhibitory synaptic input's time scale. Therefore, for the time scale of our recorded contralateral inputs, they will be more affected by inhibitory conductance than their ipsilateral counterparts. (A) Time courses for PSPs in the case of fast and slow synaptic inhibition (δ is the time that inhibition leads excitation). (B) Simulation (using the neuron model, see Methods) of PSP (EPSP + IPSP) generated with synaptic fast IPSGs having time scale of τ_inh_ = 0.1 ms and advanced with respect to the EPSGs by δ = 0.2 ms. Two different EPSGs examples: 0.15 ms (red, for ipsilateral input) or 0.25 ms (blue, for contralateral input). (C) Simulation with synaptic IPSGs with time scale of τ_inh_ = 1.0 ms and advanced with respect to the EPSGs by δ = 1.0 ms (point of full activation). Two cases: τ_exc_ = 0.15 ms (example for ipsilateral input, red), 0.25 ms (example for contralateral input, blue). For an equivalent change in synaptic inhibitory conductance (ΔGinh), the slope of the shallower EPSP displayed a greater change (arrow) than steeper EPSPs. If contralateral EPSPs have shallower rising slopes, then they are more affected by inhibition than ipsilateral EPSPs (Note: the range of total inhibitory conductance is the same in (B) and (C), since Ginh,MAX is proportional to τ_inh_).
(0.60 MB EPS)

Figure S4 **Composite EPSPs and IPSPs for an input train (500 Hz) using an idealized auditory nerve fiber model (sinusoidally modulated Poisson rate for mini-PSG times), less realistic than (Carney 1993)** [**18**] **.** (A) Raster plots with event times for ipsilateral excitatory inputs (red), contralateral excitatory inputs (blue), and contralateral inhibitory inputs (green). Superposition of composite PSGs for these three cases. Mini-EPSGs are alpha functions with time constant of 0.1 ms, and mini-IPSGs are alpha functions of 0.4 ms time const. The difference in vector strength (degree of synchrony) between the events dictates the shape of the composite EPSGs and IPSGs. Similar composite IPSGs can be generated with mini-IPSGs made of alpha functions of 0.1 ms time constant and lower vector strength (*R* = 0.48). (B) Composite PSCs corresponding to the PSGs from the final cycle of the time series in (A). EPSCs obtained with higher vector strength have steeper rising slope and shorter halfwidth (red, ipsilateral inputs; blue, contralateral inputs). IPSCs look similar to inhibitory conductance time course, because the temporal summation reached a steady state dynamic. (C) PSPs for the last cycle in (A) for the three input cases. Superimposed with a thick line is the average time course for EPSPs and IPSPs. Rising slopes are steeper when vector strength is larger (ipsilateral EPSP has steeper slope). (D) Average over 25 cycles of composite PSGs for the three different inputs. In the three cases a function (dashed) was fitted to the average PSG. Alpha function is the simplest description of PSGs (long dashes); it fits the rising phase but not the falling phase. Composite EPSGs were fitted with a functional form that is proportional to (1−(exp(−(t−t_0_)/τ_ rise_))).3.(exp(−(t−t_0_)/τ_ decay_)). Ipsilateral inputs (red): τ_rise_ = 2.5, τ_decay_ = 0.14. Contralateral inputs (blue): τ_rise_ = 2.8, τ_decay_ = 0.18. For the alpha function fits, for ipsilateral inputs (red): τ = 0.48; for contralateral inputs (blue): τ = 0.69. In the case of inhibition the best fitting was obtained with a periodic function (a.sin((t−t_0_)/n)+b), due to the time constant of the individual components and the temporal summation generated at this frequency.
(1.83 MB EPS)

## Abbreviations

CC: current clamp
EPSGs: excitatory postsynaptic conductances
ITDs: interaural time differences
LNTB: lateral nucleus of the trapezoid body
MNTB: medial nucleus of the trapezoid body
MSO: medial superior olivary neurons
PSCs: postsynaptic currents
PSPs: postsynaptic potentials
SN: strychnine
VC: voltage clamp
VCN: ventral cochlear nucleus
